# Hypertensive disorders of pregnancy: incidence, anthropometric, hemodynamic, and urinary trajectories in a Colombian cohort

**DOI:** 10.3389/fphys.2026.1856926

**Published:** 2026-07-13

**Authors:** Alejandra María Gómez-Gutiérrez, Jesús Arnulfo Velásquez-Penagos, Luis Felipe Higuita-Gutiérrez, Elkin Galeano, Diana C. Restrepo-Espinosa, Edison Osorio, Juan Carlos Quintana-Castillo, Julio César Bueno-Sánchez

**Affiliations:** 1 Grupo Reproducción, Departamento de Fisiología y Bioquímica, Facultad de Medicina, Universidad de Antioquia (UdeA), Medellín, Colombia; 2 Grupo Nacer: Salud Sexual y Reproductiva, Departamento de Ginecología y Obstetricia, Facultad de Medicina, Universidad de Antioquia (UdeA), Medellín, Colombia; 3 Facultad de Medicina, Universidad Cooperativa de Colombia (UCC), Medellín, Colombia; 4 Grupo de Investigación en Sustancias Bioactivas (GISB), Facultad de Ciencias Farmacéuticas y Alimentarias, Universidad de Antioquia (UdeA), Medellín, Colombia; 5 Red Iberoamericana de Alteraciones Vasculares en Trastornos del Embarazo (RIVATREM), Chillan, Chile

**Keywords:** blood pressure, body mass index, hypertensive disorder of pregnancy, preeclampsia, urinary creatinine

## Abstract

**Introduction:**

Hypertensive disorders of pregnancy (HDP) are among the leading causes of maternal and perinatal morbidity and mortality worldwide, particularly in low and middle-income countries. In Colombia, HDP complicate 13% of pregnancies and represent a major public health concern.

**Aim:**

To determine the incidence of HDP and characterize the longitudinal pattern of anthropometric, hemodynamic, and urinary variables in a cohort of Colombian pregnant women.

**Methods:**

A prospective cohort study was conducted including 169 pregnant women with repeated measurements of anthropometric, clinical, and urinary parameters throughout pregnancy.

**Results:**

The overall incidence of HDP was 17.8 per 100 pregnancies. Women who developed HDP delivered at a lower mean gestational age (37.3 weeks). Birth weight did not differ between groups, and neonatal length was shorter in the HDP group (median 49 cm). From early pregnancy onward, women who developed gestational hypertension had significantly higher systolic, diastolic, and mean arterial pressures (p < 0.05) compared with normotensive women. No significant differences were observed in the evolution of body mass index (BMI) trajectories or urinary biochemical markers. However, women with gestational hypertension showed a late increase in the protein/creatinine ratio.

**Conclusions:**

This study suggests subgroups of women at elevated risk for HDP in Colombia. Although these data are not fully translatable to clinical practice, they give insights into feasible preventative efforts.

## 1 Introduction

Hypertensive disorders of pregnancy (HDP) comprise heterogeneous pathological entities characterized by the new onset hypertension during gestation (≥140/90 mmHg). These disorders include: gestational hypertension, preeclampsia, superimposed preeclampsia on chronic hypertension, and eclampsia (American College of Obstetricians and Gynecologists, 2020). Collectively, HDP represent one of the leading causes of maternal and perinatal morbidity and mortality worldwide, with an incidence ranging from 4% to 25%, the highest rates occurring in low- and middle-income countries (Wang et al., 2021). In Latin America and the Caribbean, the prevalence of HDP is 9.1% (Blanco et al., 2022), with 22.1% of maternal deaths attributable to this condition (Say et al., 2014). By mid-2024, HDP were responsible for 18.9% of maternal deaths in Colombia (García, 2024), with a prevalence of 13% (Romero et al., 2022). This average obscures stark geographic disparities, in some regions, HDP account for as much as 27% of maternal mortality (Zuleta‐Tobón et al., 2013). Despite this considerable burden, the regional epidemiology of HDP remains poorly characterized. Clinical understanding has been largely extrapolated from data in high-income countries, leaving critical gaps in knowledge regarding region-specific disease patterns, long-term maternal and child health outcomes, and the trajectory to future cardiovascular disease in this population.

Most studies involving women who develop HDP describe clinical variables at a single time point, often at the end of pregnancy. However, longitudinal characterization of clinical variables would allow tracking changes throughout gestation, providing insights into causal relationships, disease progression, and temporal trends that cannot be captured in cross-sectional studies. Key clinical variables requiring close monitoring in pregnancy include body weight and body mass index (BMI). Both pre-pregnancy weight and gestational weight change can influence maternal and neonatal health. Pre-pregnancy obesity or excessive weight gain has been associated with gestational diabetes, cesarean delivery, increased blood pressure, postpartum weight retention, and fetal macrosomia (Lubrano et al., 2025). Conversely, low pre-pregnancy weight or insufficient weight gain has been linked to maternal anemia, low birth weight, and long-term health consequences in offspring (Burnie et al., 2022). Therefore, monitoring weight changes and their effects on pregnancy outcomes is crucial to improve clinical decision-making and maternal care.

Blood pressure monitoring is also essential during pregnancy. In normal gestation, blood pressure typically decreases during the first half of pregnancy due to cardiovascular and hormonal adaptations that support placental and fetal growth, then returns to pre-pregnancy values toward term (Macdonald-Wallis et al., 2012). In women with HDP, dysregulated blood pressure patterns, even within subclinical ranges, have been associated with adverse pregnancy outcomes as well as long-term blood pressure disorders and cardiovascular risks (Teng et al., 2021). Thus, evaluating blood pressure trajectories during pregnancy can provide insights into the underlying cardiovascular health of pregnant women, improve understanding of the cardiovascular adaptations occurring during gestation, and facilitate early identification of those at risk of developing hypertension.

Pregnancy induces significant renal adaptations to support increased metabolic demand. The glomerular filtration rate (GFR) rises by approximately 50%, renal plasma flow increases, and kidney size enlarges (Chandra and Paray, 2024). In healthy pregnancy, these hemodynamic changes enhance renal function without causing substantive urinary abnormalities; thus, clinically significant proteinuria or glucosuria is not considered pathological (Cheung and Lafayette, 2013). In preeclampsia (PE), one of the HDP subtypes (American College of Obstetricians and Gynecologists, 2020), both glomerular filtration rate and renal plasma flow decrease by 30%–40% compared with normal pregnancy. As a result, serum creatinine concentration, which normally decreases during gestation, fails to undergo this physiological decline and instead remain within the non-pregnant reference range (Karumanchi et al., 2005). In this context, urinalysis and measurement of urinary compounds are clinically valuable for monitoring pregnant women, as they allow detection and follow-up of maternal health alterations, particularly those affecting the kidneys and urinary tract.

This study aims to characterize the longitudinal evolution of the clinical, hemodynamic, and urinary profiles throughout pregnancy. Identifying distinct phenotypic trajectories may elucidate pathophysiological adaptation in gestation and inform targeted antenatal care for populations at highest risk of developing HDP.

## 2 Materials and methods

### 2.1 Study population

This study analyzed a nested cohort of pregnant women from the department of Antioquia (Colombia), recruited between October 2022 and October 2024. Inclusion criteria were: (a) enrollment up to 15 weeks of gestation, (b) absence of pre-existing diseases or conditions at baseline, (c) being over 18 years old, and (d) singleton pregnancy. Exclusion criteria were: (a) diagnosis of hypertension before 20 weeks of gestation, (b) twin pregnancy, (c) fetuses with malformations or chromosomal disorders, and (d) intrauterine fetal death-pregnancy loss. Additionally, participants were withdrawn from the study if they developed severe immunodeficiency, untreated bacterial or viral infection, systemic lupus erythematosus, antiphospholipid syndrome, chronic kidney disease, or active use of immunosuppressive agents during pregnancy.

#### 2.1.1 Follow-up of participants

At enrollment, each participant underwent an initial visit in which general information such as ethnicity, nationality, age (years), place of residence, educational level, gestational age (weeks), height (m), pre-pregnancy weight (kg), and blood pressure were collected. Subsequently, biweekly visits were scheduled to record weight, from which body mass index (BMI, kg/m²) was calculated. Additionally, systolic blood pressure (SBP, mmHg), diastolic blood pressure (DBP, mmHg), and mean arterial pressure (MAP, mmHg) were measured. A structured questionnaire was administered to collect data on maternal health status, routine prenatal test results, and prescribed medications. Moreover, urine samples were collected from participants at each follow-up visit. After cleansing their hands and perineum, they collected the sample (middle portion of the first urine of the day) in a sterile container. Sample adequacy was immediately confirmed via cyto-chemical assessment using a Combur Test strip (ROCHE, USA). The samples were then stored locally at -20 °C until they were sent to the University Research facilities. Upon arrival, samples were thawed, centrifuged at 1500 rpm for 10 minutes and processed for subsequent analyses.

#### 2.1.2 Study groups

The diagnosis of HDP was established based on American College of Obstetricians and Gynecologists (2020). Women who remained normotensive throughout pregnancy constituted Normotensive group. Women who developed gestational hypertension (SBP ≥140 mmHg or DBP ≥90 mmHg recorded on at least two occasions 4 h apart after 20 weeks of gestation) and women who developed preeclampsia (women who developed hypertension after 20 weeks of gestation accompanied by signs of organ involvement), were grouped under HDP. Gestational hypertension and preeclampsia were analyzed as a single HDP category for several reasons. Both conditions share overlapping clinical features, risk factors, and management strategies, and are associated with similar short- and long-term adverse outcomes including preterm birth, fetal growth restriction, and increased long-term cardiovascular risk. Moreover, evidence supports a continuum model between these entities, with gestational hypertension progressing to preeclampsia in a substantial proportion of cases (reported rates of 10–50%), particularly when onset occurs before 34 weeks of gestation. For each participant, diagnostic classification was determined using multiple sources of information, including clinical records, patient-reported information, and direct blood pressure measurements obtained during follow-up visits within the study. When elevated blood pressure values consistent with hypertension were identified during study visits, these were verified and integrated with available clinical data. When diagnostic information was uncertain or incomplete, classification was reviewed and adjudicated by at least 2 investigators. Final classification into normotensive or HDP groups was defined at the end of pregnancy follow-up. For the longitudinal analysis data from scheduled visits were consolidated into eight intervals: ≤ 10 weeks (interval 1), 11–14 weeks (interval 2), 15–18 weeks (interval 3), 19–22 weeks (interval 4), 23–26 weeks (interval 5), 27–30 weeks (interval 6), 31–34 weeks (interval 7), and ≥ 35 weeks (interval 8). Some intervals presented data loss. Data missingness was interval-specific. The gap in interval 1 reflects the enrollment window (many women enrolled in the study after week 10). Attrition in interval 8 was due to delivery or voluntary withdrawal. For intervals 2-7, missing observations resulted from logistical challenges inherent to longitudinal studies. These included participant mobility (e.g., address changes, travel), situational barriers (e.g., maternal illness, local public order concerns restricting re-searcher mobility). Importantly, this missingness was limited to specific timepoints, no subject-level attrition occurred. Time was modeled as a categorical variable (Interval, 8 levels) to reflect clinically meaningful follow-up windows during pregnancy. Intervals were primarily defined as 4-week periods; however, different intervals were used in early and late pregnancy to better capture the greater physiological variability characteristic of these stages. This approach avoids imposing a predefined functional form on time and allows for the detection of non-linear changes in the outcome across gestation. Additionally, treating time as categorical accommodates irregular measurement spacing and variability in the timing of clinical visits across participants.

### 2.2 Urinary determinations

Urinary creatinine (UCr) was measured using the Jaffé kinetic method. The creatinine reacts with picric acid in an alkaline medium to form a reddish complex known as the Janovski complex (Delanghe and Speeckaert, 2011). A commercial kit (BioSystems, Spain) was adapted for use in 96-well plates. To ensure reproducibility and precision, serial measurements were performed and monitored using Levey–Jennings charts and Westgard rules (Carroll et al., 2003), which established acceptable ranges for standards and calibrators. Briefly, 100 μL of working reagent and 10 μL of standard or urine sample were dispensed in triplicate, maintaining the 1:50 sample-to-reagent ratio specified in the manufacturer’s instructions. Absorbance readings were taken at 30 and 90 seconds after reagent addition, at a wavelength of 492 nm. Parameters such as urine specific gravity (SG) expressed as g/L and pH were determined using Combur Test strips (ROCHE) and read with the Urisys analyzer (ROCHE). In addition, urinary protein concentration (mg/L) was determined using the Bradford method, a colorimetric assay based on the shift in maximum absorbance of Coomassie Brilliant Blue G-250 from 465 nm to 595 nm upon binding to amino and carboxyl groups of proteins. Briefly, 20 μL of urine sample was mixed with 200 μL of reagent, incubated for 10 minutes, and absorbance was measured at 595 nm. A standard curve was generated using known concentrations of albumin.

### 2.3 Statistical analysis

Statistical analyses were performed using SPSS (IBM Corp.) and JASP (Version 0.95.1). Incidence rates were calculated per 100 pregnancies and compared using the Chi-square or Fisher’s exact test, as appropriate. Data distribution was assessed using the Shapiro–Wilk test. Longitudinal analyses were conducted using linear mixed-effects models (MLM) fitted by restricted maximum likelihood (REML). The models included random intercepts for subjects (ID) and fixed effects for time (Interval, treated as a categorical factor with 8 levels), Group (Normotensive vs. HDP), and the Interval × Group interaction on the continuous dependent variables. The covariance structure corresponded to compound symmetry induced by the inclusion of a random intercept and homogeneous residual variance. Random slopes for Interval and Group were not included due to insufficient within-subject variability. Degrees of freedom were estimated using the Satterthwaite approximation. Some observations were missing at specific time points; however, all 169 participants contributed data to the analyses. Missingness was considered to be mainly missing at random (MAR), and therefore all available observations were included without imputing missing values. Under the MAR assumption, REML provides unbiased estimation of fixed effects and variance components in repeated-measures longitudinal analyses. Additional MLM analyses were performed to evaluate the effect of HDP subtype and its interaction with time on the dependent variables, with subject ID included as a random effect.

## 3 Results

### 3.1 Clinical characteristics of women

A total of 175 women were enrolled in the study, of whom five were excluded due to a diagnosis of hypertension before 20 weeks of gestation, and one due to twin pregnancy. Data are therefore presented for 169 pregnant women who were followed throughout gestation. Of the women enrolled in the study, 85 (70 normotensive [50.3%] and 15 who developed HDP [50%]) joined before 11 weeks of gestation. Another 81 women (67 normotensive [48.2%] and 14 who developed HDP [46.6%]) enrolled between 11 and 14 weeks. Only 3 women enrolled at 15 weeks, of whom 2 (1.4%) remained normotensive and 1 (3.3%) developed HDP. The full distribution of gestational age at enrollment is shown in **Supplementary Table 1**. Most women in the study were of mixed race (87.5%) and Colombian nationality (96.4%). At study entry, 73.4% of participants were between 21 and 34 years of age, 66.9% had completed secondary education, and 39.1% were experiencing their first pregnancy. Regarding clinical characteristics, 56.2% were included in the study with overweight or obesity, and 19.5% were diagnosed with a urinary or vaginal infection during pregnancy. Sociodemographic results are summarized in **Table 1**.

**Table 1:** Description of the demographic and clinical characteristics of the pregnant women.

Characteristics		n	%
Ethnicity	Mestizo	148	87.5
	Afro-descendant	13	7.7
	Not defined	8	4.8
Nationality	Colombian	163	96.4
	Venezuelan	6	3.6
Age	18-20	26	15.4
	21 – 34	124	73.4
	≥35	19	11.2
Educational level	No schooling/Primary school	18	10.7
	High school	113	66.9
	University/College	38	22.5
Previous pregnancies	Primiparous	66	39.1
	Multiparous	103	60.9
BMI	Underweight	11	6.5
	Normal weight	63	37.3
	Overweight/obesity	95	56.2
Urinary tract infection/Vaginal infection	No	136	80.5
	Yes	33	19.5

### 3.2 Incidence of hypertensive disorder of pregnancy

Of the 30 women who developed HDP, 12 (40%) presented HDP before 34 weeks of gestation, indicating early-onset HDP, while 18 (60%) developed it after this gestational age, indicating late-onset HDP. The full distribution of gestational age at the development of HDP is shown in **Supplementary Table 2**. The cumulative incidence of hypertensive disorders of pregnancy (HDP) was 17.8 cases per 100 pregnancies. Higher incidences were observed among Afro-descendant women (30.8 per 100 pregnancies), Venezuelan nationals (33.3 per 100 pregnancies), and women with no formal education or only primary schooling (27.8 per 100 pregnancies). Regarding maternal age, women aged 21–34 years showed the highest incidence of HDP (19.4 per 100 pregnancies), whereas adolescents aged 20 years or younger had the lowest incidence (11.5 per 100 pregnancies). Multiparous women also showed a slightly higher incidence than primiparous women (19.4 vs. 15.2 per 100 pregnancies). According to nutritional status, HDP incidence was more than twofold higher among women with overweight or obesity than among those with normal BMI (23.2 vs. 9.5 per 100 pregnancies). In contrast, HDP incidence was similar between women with and without urinary tract or vaginal infections during pregnancy. Although higher incidences were observed across several demographic and clinical subgroups, none of the comparisons reached statistical significance (p > 0.05), possibly due to the limited sample size and the small number of events in some categories (**Table 2**).

**Table 2:** Incidence of hypertensive disorders of pregnancy according to maternal characteristics.

Characteristics		Incidence per 100 pregnant women	p-value
Ethnicity	Mestizo	16.9 (25)	0.254 ^a^
	Afro-descendant	30.8 (4)	
Nationality	Colombian	17.2 (28)	0.289 ^a^
	Venezuelan	33.3 (2)	
Age	20 years or younger	11.5 (3)	0.620 ^b^
	21 to 34 years	19.4 (24)	
	35 years or older	15.8 (3)	
Educational level	No schooling/Primary	27.8 (5)	0.470 ^b^
	High school	15.9 (18)	
	University	18.4 (7)	
Previous pregnancies	Primiparous	15.2 (10)	0.479 ^b^
	Multiparous	19.4 (20)	
BMI	Underweight	18.2 (2)	0.090 ^b^
	Normal weight	9.5 (6)	
	Overweight/Obesity	23.2 (22)	
Urinary tract infection/Vaginal infection	No	18.4 (25)	0.663 ^b^
	Yes	15.2 (5)	

^a^ Fisher’s exact test.

^b^ Pearson’s Chi-square test.

### 3.3 Follow up of anthropometric, hemodynamic, and urinary variables

To describe the longitudinal patterns associated with HDP, anthropometric, hemodynamic, and urinary variables were monitored throughout pregnancy. Although the normotensive group consisted of 139 women and the HDP group of 30 some intervals included fewer observations, as described in the methodology.

#### 3.3.1 Follow up of anthropometric variables

**Figure 1:**
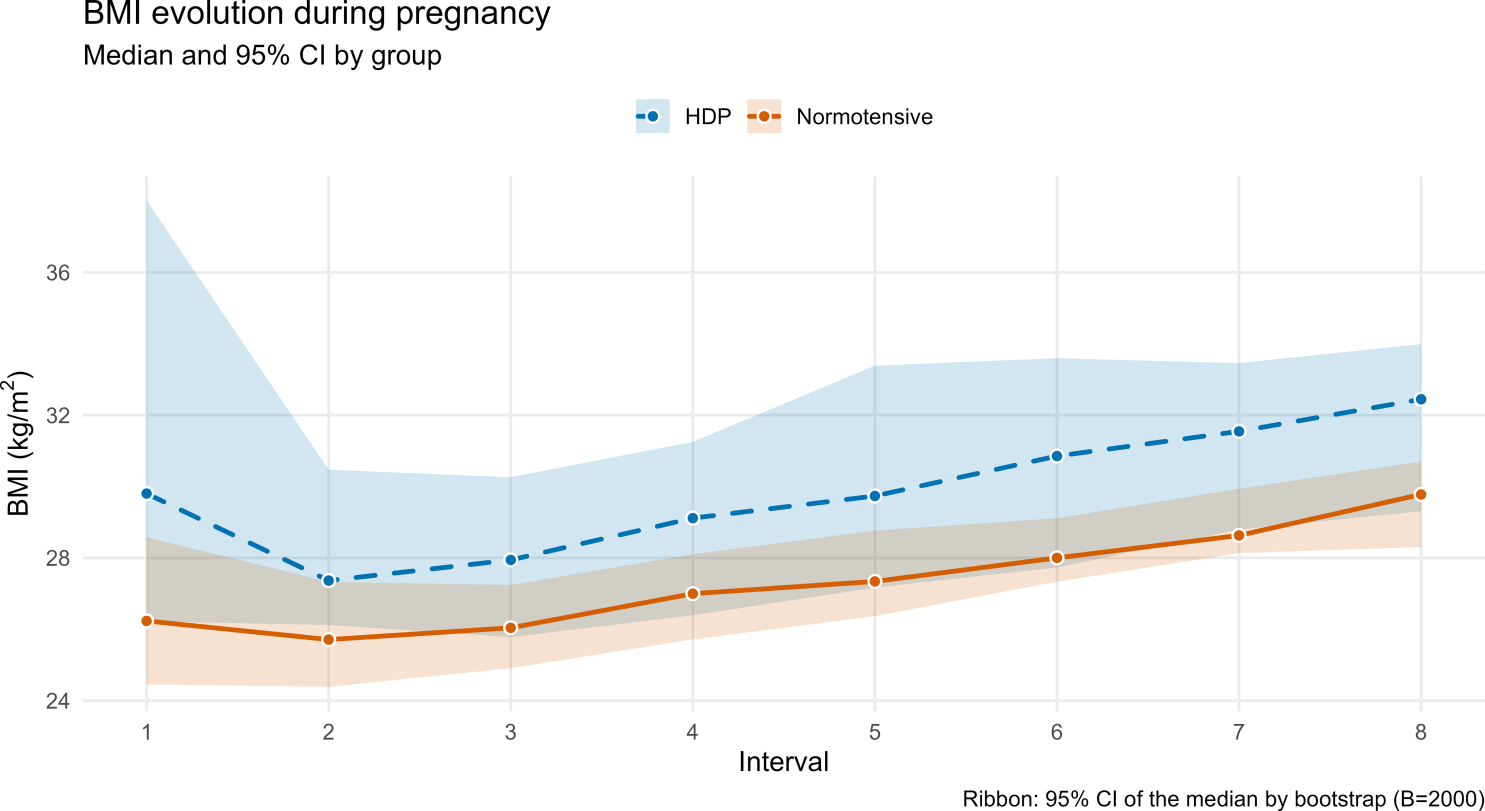
Comparison of the evolution of the body mass index at different times of pregnancy in women who develop HDP and normotensive women.

With respect to the evolution of anthropometric variables, the MLM revealed a significant effect of the interval (F = 238.96. p < 0.001), indicating substantial changes in the weight over time. A significant effect of the group was also found (F = 5.18. p = 0.024), showing systematic differences between the groups. The group and interval interaction indicates that the pattern of change over time is similar between the groups (F = 0.331. p = 0.940). A progressive increase in the weight across the intervals was observed, with the HDP group showing consistently higher values across all intervals, with an estimated average difference of +5.83 units compared to the normotensive group. The estimated change in weight per unit of time for normotensive women was 1.22 (0.74 – 1.71) kg, while for those with HDP it was 1.30 (0.25–2.34) kg (**Supplementary Table 3**). It is important to mention that total weight gain was 9 (5.5 – 11.5) kg in the normotensive group and 8.2 (5.7 – 11.4) kg in the HDP group, although results varied according to maternal weight status at study entry. Among normotensive women, those classified as underweight gained a total of 11 (7–14) kg, those with adequate weight gained 10 (9–13) kg, those who were overweight gained 7 (5– 10) kg, and those classified as obese gained 5 (3.3 – 7.8) kg. In the HDP group, underweight women gained an average of 3.5 (3 – 4) kg, women with adequate weight gained 12 (5.8 – 14) kg, overweight women gained 9 (7 – 12) kg, and obese women gained 8 (3 – 9.5) kg, and this pattern did not differ significantly between groups. BMI followed a similar trend to the weight, with a clear effect of interval (F = 230.25, p < 0.001), increasing progressively over time. Also, the HDP group exhibited consistently higher BMI values (F = 5.59, p = 0.019), and group and interval interaction indicate that the pattern of change over time is similar between the groups (F = 0.333, p = 0.939). The HDP group consistently presented higher values than the normotensive group at all intervals, with an estimated average difference of 2.28 units compared to the normotensive group. The estimated change in BMI per unit of time for normotensive women was 0.49 (0.31–0.68) kg/m², while for those with HDP it was 0.53 (0.13 – 0.93) kg/mt^2^ (**Figure 1**; **Supplementary Table 3**). We used the MLM to evaluate the effect of HDP type (early vs. late onset) on BMI, and no significant overall differences were observed between early- and late-onset HDP (F = 1,28, p = 0.172), however, a significant interaction between interval and HDP type was found (F = 7,165, p = 0.009), suggesting that the temporal evolution of the variable differs according to HDP type (data not shown).

#### 3.3.2 Follow up of hemodynamics variables

**Figure 2:**
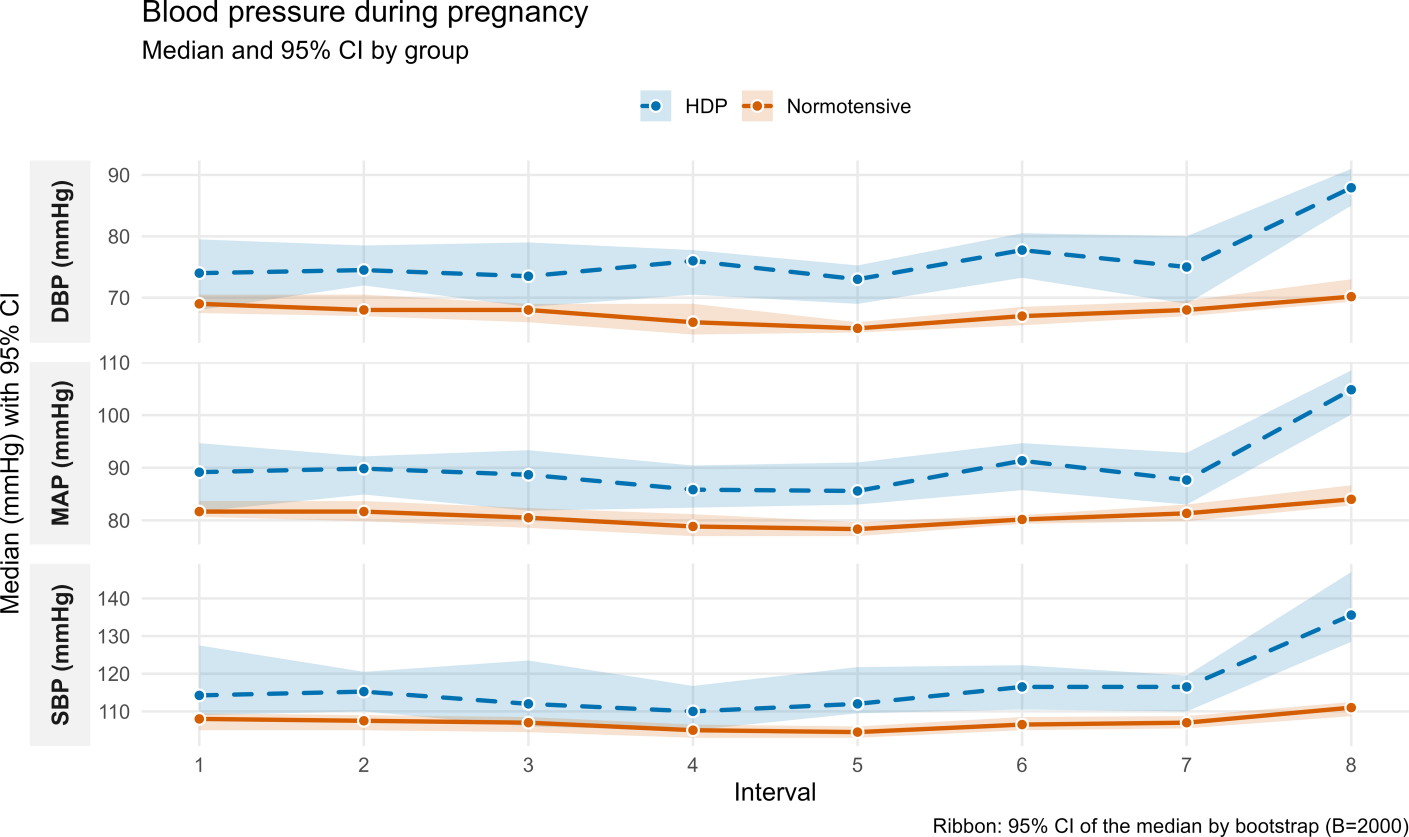
Comparison of the evolution of arterial pressure at different times of pregnancy in women who develop HDP and normotensive women.

Regarding hemodynamic variables, the results show that women who developed HDP exhibited from early pregnancy significantly higher values of SBP, DBP, and MAP compared to normotensive women (**Figure 2**). The MLM revealed a highly significant effect of time on blood pressure (SBP: F = 38.43, p < 0.001; DBP: F = 21.71, p < 0.001). Additionally, there was a statistically significant group effect, with the HDP group showing an adjusted average increase of 7.46 mmHg in SBP (F = 55.15, p < 0.001) and 2.94 mmHg in DBP (F = 51.57, p < 0.001) compared to the normotensive group. The group and interval interaction indicated that the pattern of change of blood pressure over time is different be-tween the groups (SBP: F = 15.04. p < 0.001; DBP: F = 7.24. p <0.001). The estimated change in blood pressure per unit of time for normotensive women was for SBP 0.56 (0.20 – 0.91) mmHg and DBP 0.17 (-0.11 -0.45) mmHg, while for those with HDP was for SBP 2.90 (2.12 – 3.68) mmHg and for DBP was 1.86 (1.23 – 2.49) mmHg (**Supplementary Table 4**). The MLM also revealed for blood pressure a non-linear but consistent progression over time. In the normotensive group, SBP progressively decreased from Interval 1 to Interval 4, and DBP from Interval 1 to Interval 5, followed by a return to baseline-like values by the end of gestation. In contrast, among women who developed HDP, no early-pregnancy decrease in BP was observed; instead, both SBP and DBP increased toward the end of gestation, reaching their highest values at Interval 8 (**Supplementary Table 4**). The MLM used to evaluate the effect of HDP type on SBP showed no significant overall differences between early- and late-onset HDP (F = 1,28. p = 0.096), however, a significant interaction between interval and HDP type was found (F = 7,163. p = 0.019), suggesting that the temporal evolution of the variable differs according to HDP type. This difference was particularly evident in interval 6, where patients with early-onset HDP showed higher values compared to patients with late-onset HDP. In the case of DBP, the MLM did not show significant overall differences between early- and late-onset HDP (F = 1,28. p = 0.279). Likewise, the interaction between interval and type of HDP was not significant (F = 7,163. p = 0.239), suggesting that both groups exhibit a similar temporal pattern (**Supplementary Figure 1**).

#### 3.3.3 Follow up of urinary variables

**Figure 3:**
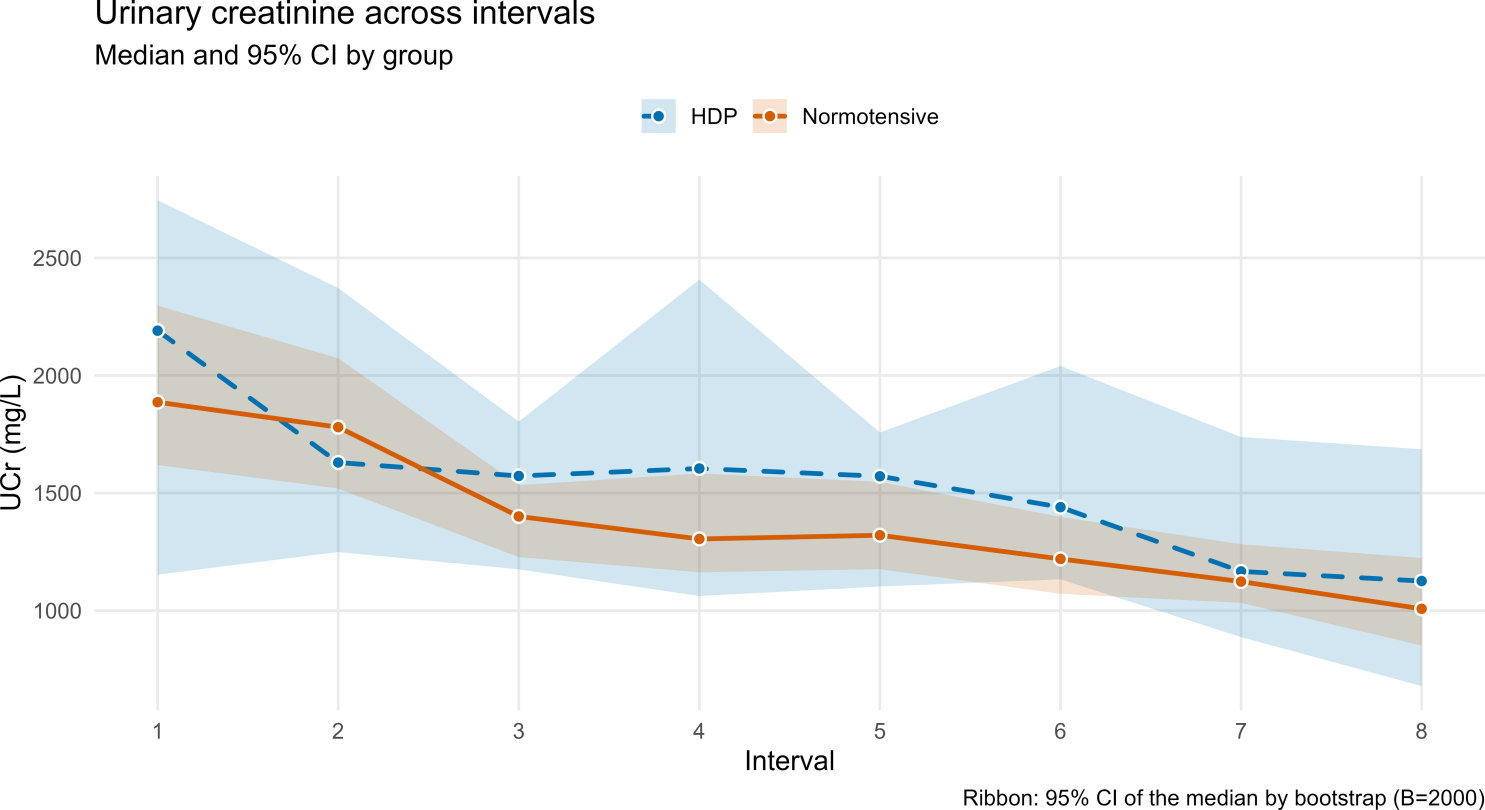
Comparison of the evolution of urinary creatinine at different stages of pregnancy in normotensive women and who develop HDP.

Urinary variables, including UCr, SG, and pH, were measured in both study groups. For UCr the MLM showed a significant effect of time (F = 5.68, p < 0.001), indicating a decrease across the analyzed intervals. However, no significant effect of group (F = 1.02, p = 0.312) or group x time interaction (F = 1.02, p = 0.413) was observed, suggesting that UCr levels evolved similarly over time in both groups. The estimated change per unit of time for normotensive women was -132 (–178 – –85) mg/L and for those with HDP was -98 (-205 – 10.5) mg/L (**Figure 3**; **Supplementary Table 5**). For SG, a modest decrease across intervals was also observed (F = 7.98, p < 0.001). No significant effect of group (F = 0.26, p = 0.609) or group × interval interaction (F = 1.06, p = 0.384) was found, indicating similar trajectories in both groups throughout gestation (**Supplementary Table 5**). For urinary pH, values increased progressively across intervals (F = 2.29, p = 0.025), with no significant group effect or interaction, suggesting preserved renal acid–base homeostasis in both groups (**Supplementary Table 5**). An MLM was used to evaluate the effect of HDP type on UCr. No significant effects of HDP type were observed (F = 1,28, p = 0.161). Likewise, the interaction between interval and HDP type was not significant (F = 7,153, p = 0.191), suggesting a similar temporal pattern between both groups. High interindividual and residual variability in the measurements was observed (data not shown).

An analysis of proteinuria was performed with a subgroup of 42 women (21 Normotensive vs 21 HDP) that was matched by ethnicity, age, BMI, and mode of delivery. Since creatinine excretion is considered constant over a 24-hour period and correlates well with total 24-hour protein excretion, we determined the protein-to-creatinine ratio (PCR) to assess renal function. This index is calculated from protein and creatinine concentrations as follows: PCR = Protein (mg)/Creatinine (mmol). The MLM revealed significant main effect of interval (F = 2.30, p = 0.028) and a significant interval × Group interaction (F = 2.07, p = 0.047) in PCR, while the main effect of Group was not significant (F = 2.27, p = 0.141). In summary, both groups followed a comparable trajectory over time, but women with HDP showed a marked increase in the final interval 6.31 (4.19 - 8.44) mg/mmol compared with normotensive women 0.37 (–1.64 - 2.38) mg/mmol, with an estimated difference of 5.73 units (**Supplementary Figure 2**, **Supplementary Table 6**).

#### 3.3.4 Other pregnancy outcomes

Finally, pregnancy outcomes including gestational age at delivery, mode of delivery, birth weight, and neonatal length were evaluated. Gestational age at delivery (p < 0.001) and length of neonates (p = 0.048) were significantly lower in women with HDP (**Table 3**). In addition, cesarean delivery was more frequent in the HDP group (**Table 4**). It is also noteworthy that approximately 30% of the women in the study developed other complications, with gestational diabetes mellitus being the most common in HDP women and anemia in normotensive women (data not shown).

**Table 3:** Delivery week, newborn size, and weight in women with and without HDP.

Variable	Normotensive		HDP		p-value
	Median	IQR	Median	IQR	
Gestational age at delivery	39.0	38.0-39.6	37.3	36.4-39.0	<0.001
Neonate weight	3190.0	2997.0-3400	3100	2680-3430	0.330
Neonate length	50.0	48.5-51.0	49.0	48.0-50.0	0.048

Interquartile Range (IQR). Mann–Whitney U.

**Table 4:** Type of delivery in women with and without HDP.

Variable	Normotensive		HDP		p-value
	n	%	n	%	
Vaginal birth	81	58.3	9	36	0.039
Cesarean delivery	58	41.7	16	64	

Chi-square test.

## 4 Discussion

This study provides the first longitudinal characterization of clinical variables throughout pregnancy in a socioeconomically vulnerable Colombian cohort. The observed incidence of HDP was 17.8 per 100 pregnancies, with higher rates observed among Afro-Colombian women (30.8), women with low educational attainment (27.8), and those with overweight or obesity (23.2). This elevated incidence aligns with the current literature, which reports a rising global HDP incidence of 10–15% over the past 30 years, particularly in regions with low sociodemographic indices (Mhiri et al., 2020; Wang et al., 2021). Socioeconomic status is a well-established determinant, as women with HDP frequently have low household income and limited access to essential healthcare (Sun et al., 2025). The study was conducted in the regions of Bajo Cauca and Urabá, where 87.9% and 86% of households, respectively, experience food insecurity (Universidad de Antioquia et al., 2019). This context is closely linked to low purchasing power and educational access, which constrain nutritional adequacy and maternal health. Additionally, the rising prevalence of risk factors such as obesity and metabolic syndrome may partly explain the incidence of HDP observed in this study (Lazzari et al., 2025). Notably, 56% of participants entered pregnancy with overweight or obesity. Previous research indicates that excessive pregestational weight increases the risk of preeclampsia, gestational diabetes, fetal macrosomia, and postpartum weight retention (Kominiarek and Peaceman, 2017). These findings highlight the greater health risks faced by women in regions marked by profound social inequalities, such as those from which participants were recruited (Universidad de Antioquia et al., 2019).

Women with HDP consistently showed higher values of weight and BMI. This is relevant because both maternal overweight and excessive gestational weight gain have been associated with pro-inflammatory states. These alterations have been associated with insulin resistance, predisposing mothers to metabolic disturbances during pregnancy (as observed in the cohort) or to an increased risk of such complications later in life (Parrettini et al., 2020; Sun et al., 2020). In this study, no differences were found in total gestational weight gain between normotensive and HDP women with values of 0.75 kg for the first trimester, 5.85 kg for the second, and 9 kg for the third. These results are comparable to those reported in an Indian cohort (8,235 women), who exhibited gains of 1.29 kg at week 18, 4.44 kg at week 26, and 9.06 kg at week 40 (Thiruvengadam et al., 2022). Weight gain trajectories and cumulative weight gain according to BMI classification at study entry were described, and their association with neonatal weight was analyzed. It was observed that weight gain trajectories varied across BMI categories, indicating that pregestational BMI strongly conditions gestational weight trajectories. Since 2016, Colombia has adopted the recommendations by Atalah et al. (1997) as the national reference for maternal weight gain. These guidelines suggest a gain of 12–18 kg for underweight women, 10–13 kg for those with normal BMI, 7–10 kg for overweight women, and 6–7 kg for women with obesity. Such ranges aim to reduce maternal risks and support adequate fetal development and optimal birthweight. Enrolled women met these recommended ranges, except for underweight women with HDP (who gained less than expected), and obese women with HDP (who gained slightly more). Furthermore, in normotensive women, neonates achieved adequate birthweight and length regardless of maternal BMI classification. However, in the HDP group, although the overall median birthweight was not significantly different from that of normotensive women, neonates of mothers with normal BMI and underweight were classified as low birthweight and insufficient birthweight, respectively. In addition, neonates born to hypertensive mothers had shorter length at birth. These findings indicate that HDP disrupts adequate fetal growth and reinforce previous reports linking HDP, particularly preeclampsia, with low birthweight (Liu et al., 2021).

Pregnancy entails profound cardiovascular adaptations, including a physiological decrease in peripheral vascular resistance during the first half of gestation, which reaches its nadir by mid-second trimester and is followed by stabilization or a mild increase thereafter (Sanghavi and Rutherford, 2014). The clinical trajectory of normotensive women aligned with expected patterns during follow-up, whereas those who developed hypertensive disorders exhibited consistently higher blood pressure values—even before clinical onset—suggesting an altered baseline hemodynamic state. In hypertensive pregnancies, systolic blood pressure showed minimal early variation and a late third-trimester rise, while diastolic pressure demonstrated only minor reductions and an earlier rebound compared with normotensive women. These observed patterns, while not identical, are broadly consistent with the meta-analysis by Haas et al., which confirmed a characteristic lack of systolic decline and an exaggerated third-trimester rise in blood pressure in hypertensive pregnancies (de Haas et al., 2022). Taken together, these findings are consistent with — though do not establish — the hypothesis that HDP may reflect impaired vascular adaptation. Specifically, the absent systolic decline and premature diastolic rebound are associated with patterns previously described in states of reduced arterial compliance and heightened vascular tone, although the present data do not allow direct assessment of vascular mechanisms. These observations are further compatible with the hypothesis that subclinical cardiovascular vulnerability may already be present before conception, potentially limiting the maternal circulatory system’s adaptive capacity under the hemodynamic demands of pregnancy (Fang et al., 2025); however, this interpretation remains speculative given the observational nature of the study. These hemodynamic findings hold direct clinical relevance, as they may represent the early physiological underpinnings of a higher-risk pregnancy trajectory. The 2017 American College of Cardiology/American Heart Association (ACC/AHA) guidelines, which define stage 1 hypertension as 130–139/80–89 mmHg, have identified a novel, intermediate-risk population of pregnant women when this condition is present in early pregnancy (≤13 weeks). Current evidence consistently associates early stage 1 hypertension with a significantly elevated risk for PE, gestational diabetes, and indicated preterm birth compared to normotensive women (Horgan et al., 2024). Consequently, the ACC/AHA classification appears to provide superior risk assessment capability at the first prenatal visit compared to the traditional obstetric threshold of ≥140/90 mmHg for diagnosing chronic hypertension (Horgan et al., 2025).

The current study identified a decreasing trend in UCr and SG concentrations across gestation in both groups of women. While this phenomenon has previously been reported in spot urine samples from normotensive and preeclamptic women (Kuromoto et al., 2010; Bu et al., 2021), to our knowledge, this is the first time it is documented in Colombia. The precise mechanisms underlying these findings remain incompletely understood, but adaptations in the renin–angiotensin–aldosterone system (RAAS) are likely involved. Increased angiotensinogen activity raises angiotensin II levels from early pregnancy onwards (Beers and Patel, 2020), promoting sodium and, more importantly, water retention (estimated at 6–8 L), including (~280 vs. 289 mOsm/L) (Costantine, 2014), which can lead to less concentrated urine and may explain the progressive reduction of UCr and SG observed in the cohort. According to current criteria, urine samples with CrU <470 mg/L are considered dilute in pregnancy (Baba et al., 2017), whereas values >3000 mg/L indicate concentrated urine (Mikheev and Lowry, 1996). In this case, the frequency of dilute urines increased with advancing gestation (4.0%, 11.7%, and 28.4% across trimesters), while concentrated samples declined (28.3%, 19.8%, 13.2%). Interestingly, none of the 35 nonpregnant women studied exhibited dilute urine (data not shown). These outcomes reinforce the notion that gestation-associated plasma volume expansion drives urine dilution, independently of hypertensive status. Urinary pH remained stable across groups and gestational intervals, consistent with preserved renal acid–base homeostasis. The mean urinary pH (~6) observed is physiologically optimal, ensuring solubility of uric acid and phosphate salts, thus minimizing risk of uric acid or calcium phosphate stone formation (Worcester et al., 2018). Notably, in a matched subgroup analysis, an increase in proteinuria (within the normal range) and a corresponding rise in PCR we detected at late gestation in HDP women but not in normotensives. Since PCR correlates well with 24-h protein excretion and a threshold of 30 mg/mmol is clinically accepted for significant proteinuria (Côté et al., 2008), these observations do not support overt renal damage. Nevertheless, the higher PCR values in the HDP group could suggest mild pregnancy-related changes in renal permeability or protein handling, although no direct evidence of renal injury was identified in this study (O and S, 2019). It is also possible that these findings reflect early pregnancy-related renal adaptations previously described in HDP, including alterations in glomerular endothelial or podocyte integrity; however, this study was not designed to evaluate these mechanisms directly (Fishel Bartal et al., 2022).

Although this study offers novel insights into the physiological trajectories associated with HDP in a vulnerable population, several limitations must be acknowledged. The absence of significant differences in some comparisons may have been influenced by the moderate statistical power observed for certain anthropometric variables and the lower power for some urinary variables, increasing the possibility of Type II error. This limitation was mainly related to the relatively small number of HDP cases (n = 30) (Horgan et al., 2024), interval-specific missing data, and the inherent biological variability of these measures. Missingness was primarily associated with logistical or clinical circumstances occurring at specific visits rather than systematic participant dropout, suggesting mechanisms compatible with MCAR/MAR assumptions. Accordingly, linear mixed-effects models fitted using REML were considered appropriate, as they allow the inclusion of all available repeated measurements without requiring complete follow-up for every participant. Nevertheless, the reduced sample size in some intervals may have decreased the precision of certain trajectory estimates. Despite this, significant differences were still identified in several analyses, supporting the robustness of those findings. We also emphasize that these associations should be interpreted as exploratory rather than explanatory or causal regarding HDP. In contrast, blood pressure variables consistently showed p values < 0.05 across several intervals, supported by high statistical power (>0.80), indicating a lower probability of Type II error for these outcomes. Furthermore, the specific demographic profile of our participants (predominantly from socioeconomically vulnerable urban and peri-urban areas) may limit the generalizability of the findings. The relationships described here may differ in populations with higher socioeconomic status, different ethnic composition, or better healthcare access. Finally, while confounding variables likely exist, the descriptive nature of this study precluded formal adjustment, aligning with its primary aim of characterization rather than causal inference. Furthermore, the joint analysis of gestational hypertension and preeclampsia under a single HDP category, while justified by clinical and epidemiological evidence, may mask subtype-specific differences in trajectory patterns that could be detectable in larger samples. These findings must be interpreted within the broader regional context, a perspective compellingly articulated by Giachini, et al (Giachini et al., 2017). As they highlight, in Latin-American countries where PE is one of the leading causes of maternal and fetal mortality, information about this disease has been mainly extrapolated from studies conducted in developed countries. This means that knowledge about regional particularities of the disease and its consequences in mothers and their children—or even the relationship to future cardiovascular disease—remains critically limited. Latin America presents profound cultural, sociodemographic, economic, and geographic heterogeneity, which must be central to any epidemiological analysis. While molecular mechanisms and early screening in high-income countries are at the forefront of PE research, our study addresses a prior, fundamental gap identified by Giachini et al.: establishing the phenotypic expression and trajectories of HDP in vulnerable Latin American populations. This foundational epidemiological work is a prerequisite for the valid adaptation of advanced diagnostics and the development of effective, region-specific prevention strategies. As Giachini and colleagues argue, there is a crucial need for collaborative research efforts across the region to broaden the search for new strategies directed to the understanding of vascular disorders of pregnancy to lead to improved health outcomes in our countries.

## 5 Conclusion

Results showed that women who developed HDP had higher BMI and blood pressure values from early pregnancy, along with an altered hemodynamic pattern characterized by the absence of the physiological second-trimester blood pressure decline and a greater increase toward late pregnancy. While both groups showed progressive reductions in urinary creatinine and specific gravity consistent with physiological dilution, women with HDP exhibited a late increase in PCR; while this observation is exploratory and values remained within physiological ranges, it may warrant further investigation as a potential marker of early renal adaptation in HDP. These findings indicate a distinct HDP risk profile in this vulnerable population, characterized by marked social inequities and high obesity prevalence, underscoring the need for improved nutrition, weight control, prenatal care, and larger collaborative studies across Latin America to guide prevention and management strategies. Such coordinated initiatives are essential to validate these exploratory findings, elucidate causal pathways, and ultimately improve health outcomes for mothers and children affected by hypertensive pregnancy disorders.

## Data Availability

The raw data supporting the conclusions of this article will be made available by the authors, without undue reservation.
