# Supplementary material for: Hypertensive disorders of pregnancy: incidence, anthropometric, hemodynamic, and urinary trajectories in a Colombian cohort
Source: Front Physiol. 2026 Jul 13;17:1856926. doi: 10.3389/fphys.2026.1856926 (PMC13402200; doi:10.3389/fphys.2026.1856926)

Supplementary Material

**Supplementary Table 1. Distribution of gestational age at enrollment**

|  | ≤ 10 Weeks  n = 85 | 11 – 14 Weeks  n = 81 | Week 15  n = 3 |
| --- | --- | --- | --- |
| Mean ± SD | 8.24 ± 1.52 | 12.6 ± 1.2 | 15 ± 0 |
| Median  (IQR) | 8  (7 – 9) | 13  (11 – 14) | 15  (15 – 15) |

Standard deviation (SD); interquartile range (IQR).

**Supplementary Table 2. Distribution of gestational age at HDP onset**

|  | < 34 weeks  n = 12 | ≥ 34 weeks  n = 18 |
| --- | --- | --- |
| Mean ± SD | 27.6 ± 3.17 | 36.3 ± 1.37 |
| Median  (IQR) | 27  (23.8 – 28.8) | 36  (35.8 – 37.3) |

Standard deviation (SD); interquartile range (IQR).

**Supplementary Table 3. Changes in maternal anthropometric variables throughout pregnancy**

|  |  | **Normotensive** | | | | | | **HDP** | | | | | |
| --- | --- | --- | --- | --- | --- | --- | --- | --- | --- | --- | --- | --- | --- |
| **Variable** | **Int.** | **n** | **Extent of missing data %** | **Est.** | **SE** | **95% CI**  **Lower - Upper** | **p** | **n** | **Extent of missing data %** | **Est.** | **SE** | **95% CI**  **Lower - Upper** | **p** |
| Weight (Kg) | 1 | 69 | 50.4 | 66.12 | 1.22 | 63.7 – 68.5 | **<0.001** | 14 | 53.4 | 71.96 | 2.64 | 66.7 – 77.1 | **0.047** |
|  | 2 | 133 | 4.4 | 65.99 | 1.21 | 63.6 – 68.3 | 0.644 | 30 | 0 | 72.60 | 2.61 | 67.4 – 77.7 | 0.266 |
|  | 3 | 137 | 1.5 | 66.77 | 1.21 | 64.3 – 69.1 | **0.025** | 29 | 3.4 | 73.52 | 2.61 | 68.3 – 78.6 | 0.195 |
|  | 4 | 136 | 2.2 | 68.50 | 1.2 | 66.1 – 70.8 | **<0.001** | 30 | 0 | 75.13 | 2.61 | 70 – 80.2 | 0.256 |
|  | 5 | 135 | 2.9 | 70.15 | 1.21 | 67.7 – 72.5 | **<0.001** | 28 | 6.7 | 76.75 | 2.61 | 71.6 – 81.8 | 0.276 |
|  | 6 | 135 | 2.9 | 71.64 | 1.21 | 69.2 – 74 | **<0.001** | 29 | 3.4 | 78.23 | 2.61 | 73.1 – 83.4 | 0.244 |
|  | 7 | 131 | 5.8 | 73.26 | 1.21 | 70.8 – 75.6 | **<0.001** | 26 | 13.4 | 80.02 | 2.62 | 74.8 – 85.1 | 0.198 |
|  | 8 | 119 | 14.4 | 74.68 | 1.21 | 72.2 – 77 | **<0.001** | 23 | 23.4 | 81.04 | 2.62 | 75.8 – 86.1 | 0.478 |
| BMI (Kg/mt^2^) | 1 | 69 | 50.4 | 26.39 | 0.47 | 25.4 – 27.3 | **<0.001** | 14 | 53.4 | 28.68 | 1.01 | 26.6 – 30.6 | **0.042** |
|  | 2 | 133 | 4.4 | 26.32 | 0.46 | 25.4 – 27.2 | 0.570 | 30 | 0 | 28.94 | 1 | 26.9 – 30.8 | 0.256 |
|  | 3 | 137 | 1.5 | 26.64 | 0.46 | 25.7 – 27.5 | **0.035** | 29 | 3.4 | 29.31 | 1 | 27.3 – 31.2 | 0.183 |
|  | 4 | 136 | 2.2 | 27.33 | 0.46 | 26.4 – 28.2 | **<0.001** | 30 | 0 | 29.96 | 1 | 28 – 31.9 | 0.240 |
|  | 5 | 135 | 2.9 | 28 | 0.46 | 27 – 28.9 | **<0.001** | 28 | 6.7 | 30.61 | 1 | 28.6 – 32.5 | 0.266 |
|  | 6 | 135 | 2.9 | 28.59 | 0.46 | 27.6 – 29.5 | **<0.001** | 29 | 3.4 | 31.24 | 1 | 29.2 – 33.2 | 0.215 |
|  | 7 | 131 | 5.8 | 29.25 | 0.46 | 28.3 – 30.1 | **<0.001** | 26 | 13.4 | 31.93 | 1 | 29.9 – 33.8 | 0.193 |
|  | 8 | 119 | 14.4 | 29.83 | 0.46 | 28.9 – 30.7 | **<0.001** | 23 | 23.4 | 32.36 | 1 | 30.3 – 34.3 | 0.419 |

Int. = interval; Est. = estimate; SE = standard error.

P-values correspond to the fixed effects of the linear mixed model (LMM) and were estimated using Satterthwaite’s approximation. They test whether each term (intercept, interval, group, and their interactions) differs significantly from zero or from the reference category.

These comparisons are exploratory in nature; the absence of significant differences in some cases does not necessarily indicate a true lack of effect, as the statistical power was below 80% for some analyses.

**Supplementary Table 4. Changes in hemodynamic variables throughout pregnancy**

|  |  | **Normotensive** | | | | | | **HDP** | | | | | |
| --- | --- | --- | --- | --- | --- | --- | --- | --- | --- | --- | --- | --- | --- |
| **Variable** | **Int.** | **n** | **Extent of missing data %** | **Est.** | **SE** | **95% CI**  **Lower - Upper** | **p** | **n** | **Extent of missing data %** | **Est.** | **SE** | **95% CI**  **Lower - Upper** | **p** |
| SBP (mmHg) | 1 | 69 | 50.4 | 108.1 | 0.97 | 106.2 – 110 | **<0.001** | 14 | 53.4 | 115.6 | 2.14 | 111.4 – 119.8 | **0.002** |
|  | 2 | 133 | 4.4 | 107.9 | 0.77 | 106.4 – 109.5 | 0.871 | 30 | 0 | 113.9 | 1.64 | 110.6 – 117.1 | 0.523 |
|  | 3 | 138 | 0.8 | 107.3 | 0.76 | 105.8 – 108.8 | 0.400 | 29 | 3.4 | 113.9 | 1.66 | 110.7 – 117.2 | 0.743 |
|  | 4 | 137 | 1.5 | 105.5 | 0.76 | 104 – 107 | **0.010** | 30 | 0 | 112.6 | 1.64 | 109.4 – 115.8 | 0.868 |
|  | 5 | 135 | 2.9 | 105.4 | 0.77 | 103.9 – 106.9 | **0.007** | 28 | 6.7 | 114.9 | 1.68 | 111.6 – 118.2 | 0.407 |
|  | 6 | 135 | 2.9 | 107.6 | 0.77 | 106.1 – 109.1 | 0.603 | 28 | 6.7 | 119.1 | 1.68 | 115.8 – 122.4 | 0.094 |
|  | 7 | 130 | 6.5 | 108 | 0.78 | 106.5 – 109.6 | 0.933 | 25 | 16.7 | 115.7 | 1.74 | 112.3 – 119.1 | 0.938 |
|  | 8 | 116 | 16.6 | 112 | 0.80 | 110.4 – 113.6 | **<0.001** | 22 | 26.7 | 135.9 | 1.82 | 132.4 – 139.5 | **<0.001** |
| DBP (mmHg) | 1 | 69 | 50.4 | 70.48 | 0.78 | 68.9 – 72 | **<0.001** | 14 | 53.4 | 73.42 | 1.72 | 70 – 76.8 | 0.121 |
|  | 2 | 133 | 4.4 | 69.72 | 0.61 | 68.5 – 70.9 | 0.356 | 30 | 0 | 74.57 | 1.3 | 72 – 77.1 | 0.338 |
|  | 3 | 138 | 0.8 | 68.23 | 0.60 | 67 – 69.4 | **0.006** | 29 | 3.4 | 73.72 | 1.32 | 71.1 – 76.3 | 0.201 |
|  | 4 | 137 | 1.5 | 66.56 | 0.61 | 65.3 – 67.7 | **<0.001** | 30 | 0 | 74.3 | 1.3 | 71.7 – 76.8 | **0.016** |
|  | 5 | 135 | 2.9 | 65.99 | 0.61 | 64.7 – 67.1 | **<0.001** | 28 | 6.7 | 72.93 | 1.33 | 70.3 – 75.5 | **0.046** |
|  | 6 | 135 | 2.9 | 67.52 | 0.61 | 66.3 – 68.7 | **<0.001** | 28 | 6.7 | 76.73 | 1.33 | 74.1 – 79.3 | **0.002** |
|  | 7 | 130 | 6.5 | 68.89 | 0.62 | 67.6 – 70.1 | 0.055 | 25 | 16.7 | 76.24 | 1.39 | 73.5 – 78.9 | **0.031** |
|  | 8 | 116 | 16.6 | 71.69 | 0.64 | 70.4 – 72.9 | 0.154 | 22 | 26.7 | 86.42 | 1.45 | 83.5 – 89.2 | **<0.001** |

Int. = interval; Est. = estimate; SE = standard error.

P-values correspond to the fixed effects of the linear mixed model (LMM) and were estimated using Satterthwaite’s approximation. They test whether each term (intercept, interval, group, and their interactions) differs significantly from zero or from the reference category.

**Supplementary Figure 1.** Comparison of blood pressure (BP) changes across the stages of pregnancy between women with early-onset and late-onset HDP.

**Supplementary Table 5. Changes in maternal urinary variables throughout pregnancy**

|  |  | **Normotensive** | | | | | | **HDP** | | | | | |
| --- | --- | --- | --- | --- | --- | --- | --- | --- | --- | --- | --- | --- | --- |
| **Variable** | **Int.** | **n** | **Extent of missing data %** | **Est.** | **SE** | **95% CI**  **Lower - Upper** | **p** | **n** | **Extent of missing data %** | **Est.** | **SE** | **95% CI**  **Lower - Upper** | **p** |
| UCr (mg/L) | 1 | 68 | 51.1 | 2255 | 130.2 | 1999.2 – 2510 | **<0.001** | 13 | 56.7 | 2147 | 300.5 | 1558.4 – 2737 | 0.744 |
|  | 2 | 128 | 8 | 2106 | 100 | 1909.5 – 2302 | 0.315 | 28 | 6.7 | 2109 | 214.8 | 1688.3 – 2531 | 0.763 |
|  | 3 | 132 | 5.1 | 1597 | 98.4 | 1404.1 – 1790 | **<0.001** | 29 | 3.4 | 2056 | 211.9 | 1640.6 – 2472 | 0.122 |
|  | 4 | 132 | 5.1 | 1589 | 98.4 | 1396.3 – 1782 | **<0.001** | 28 | 6.7 | 2073 | 211.9 | 1657.5 – 2488 | 0.106 |
|  | 5 | 131 | 5.8 | 1629 | 99 | 1434.6 – 1823 | **<0.001** | 27 | 10 | 1609 | 218.4 | 1180.9 – 2037 | 0.813 |
|  | 6 | 133 | 4.4 | 1526 | 98.7 | 1332.5 – 1720 | **<0.001** | 26 | 13.4 | 1703 | 221.9 | 1268 – 2138 | 0.443 |
|  | 7 | 124 | 10.8 | 1462 | 101 | 1264.3 – 1660 | **<0.001** | 23 | 23.4 | 1500 | 233.9 | 1041.7– 1958 | 0.703 |
|  | 8 | 110 | 20.9 | 1332 | 105.7 | 1124.3 - 1539 | **<0.001** | 19 | 36.7 | 1463 | 242.9 | 986.9 - 1939 | 0.537 |
| SG (g/L) | 1 | 70 | 49.7 | 1.016 | 0.54 | 1.015 – 1.017 | **<0.001** | 13 | 56.7 | 1.016 | 1.26 | 1.013 – 1.018 | 0.682 |
|  | 2 | 123 | 11.6 | 1.015 | 0.44 | 1.014 – 1.016 | 0.061 | 27 | 10 | 1.017 | 0.94 | 1.015 – 1.019 | 0.155 |
|  | 3 | 130 | 6.5 | 1.015 | 0.43 | 1.014 – 1.016 | **0.008** | 23 | 23.4 | 1.016 | 1 | 1.014 – 1.018 | 0.255 |
|  | 4 | 131 | 5.8 | 1.013 | 0.43 | 1.012 – 1.014 | **<0.001** | 25 | 16.7 | 1.015 | 0.97 | 1.013 – 1.017 | 0.167 |
|  | 5 | 125 | 10.1 | 1.014 | 0.43 | 1.013 – 1.015 | **<0.001** | 24 | 20 | 1.014 | 0.99 | 1.012 – 1.016 | 0.813 |
|  | 6 | 125 | 10.1 | 1.014 | 0.43 | 1.013 – 1.015 | **<0.001** | 25 | 16.7 | 1.013 | 0.97 | 1.011 – 1.015 | 0.983 |
|  | 7 | 111 | 20.2 | 1.013 | 0.45 | 1.012 – 1.014 | **<0.001** | 21 | 30 | 1.013 | 1 | 1.011 – 1.015 | 0.809 |
|  | 8 | 103 | 25.9 | 1.013 | 0.47 | 1.012 – 1.014 | **<0.001** | 17 | 43.4 | 1.012 | 1.13 | 1.010 – 1.014 | 0.901 |
| pH | 1 | 70 | 49.7 | 6.2 | 0.08 | 6.0 – 6.3 | **<0.001** | 13 | 56.7 | 5.99 | 0.18 | 5.6 – 6.3 | 0.298 |
|  | 2 | 123 | 11.6 | 6.32 | 0.06 | 6.1 – 6.4 | 0.209 | 27 | 10 | 6.06 | 0.13 | 5.7 – 6.3 | 0.834 |
|  | 3 | 130 | 6.5 | 6.37 | 0.06 | 6.2 – 6.4 | 0.078 | 23 | 23.4 | 6.12 | 0.14 | 5.8 – 6.4 | 0.870 |
|  | 4 | 131 | 5.8 | 6.5 | 0.06 | 6.3 – 6.6 | **0.001** | 25 | 16.7 | 6.17 | 0.14 | 5.8 – 6.4 | 0.596 |
|  | 5 | 125 | 10.1 | 6.37 | 0.06 | 6.2 – 6.5 | 0.068 | 24 | 20 | 6.30 | 0.14 | 6 – 6.5 | 0.542 |
|  | 6 | 125 | 10.1 | 6.44 | 0.06 | 6.3 – 6.5 | **0.011** | 25 | 16.7 | 6.38 | 0.14 | 6.1 – 6.6 | 0.502 |
|  | 7 | 111 | 20.2 | 6.51 | 0.06 | 6.3 – 6.6 | **0.002** | 21 | 30 | 6.38 | 0.15 | 6 – 6.6 | 0.699 |
|  | 8 | 103 | 25.9 | 6.44 | 0.07 | 6.3 – 6.5 | **0.017** | 17 | 43.4 | 6.28 | 0.17 | 5.9 – 6.6 | 0.832 |

Int. = interval; Est. = estimate; SE = standard error.

P-values correspond to the fixed effects of the linear mixed model (LMM) and were estimated using Satterthwaite’s approximation. They test whether each term (intercept, interval, group, and their interactions) differs significantly from zero or from the reference category.

These comparisons are exploratory in nature; the absence of significant differences in some cases does not necessarily indicate a true lack of effect, as the statistical power was below 80% for some analyses.

**Supplementary Table 6. Changes in the protein-to-creatinine ratio (PCR) throughout pregnancy**

|  |  | **Normotensive** | | | | | | **HDP** | | | | | |
| --- | --- | --- | --- | --- | --- | --- | --- | --- | --- | --- | --- | --- | --- |
| **Variable** | **Int.** | **n** | **Extent of missing data %** | **Est.** | **SE** | **95% CI**  **Lower - Upper** | **p** | **n** | **Extent of missing data %** | **Est.** | **SE** | **95% CI**  **Lower - Upper** | **p** |
| PCR (mg/mmol) | 1 | 8 | 62 | 0 | 0.10 | -2.9 – 3.1 | 0.949 | 10 | 52.4 | 1 | 0.31 | -2.4 – 3 | 0.921 |
|  | 2 | 20 | 4.8 | 0 | 0.06 | -1.8 – 2 | 0.985 | 19 | 9.6 | 1 | 0.21 | -1.7 – 2.2 | 0.980 |
|  | 3 | 21 | 0 | 0 | 0.19 | -1.7 – 2.1 | 0.958 | 18 | 14.3 | 1 | 0.15 | -1.9 – 2.2 | 0.921 |
|  | 4 | 21 | 0 | 0 | 0.25 | -1.6 – 2.1 | 0.934 | 21 | 0 | 1 | 0.31 | -1.5 – 2.2 | 0.953 |
|  | 5 | 20 | 4.8 | 0 | 0.56 | -1.3 – 2.5 | 0.799 | 18 | 14.3 | 1 | 0.62 | -1.4 – 2.6 | 0.952 |
|  | 6 | 19 | 9.6 | 0 | 0.61 | -1.3 – 2.6 | 0.782 | 19 | 9.6 | 1 | 2 | 0.06 – 4 | 0.619 |
|  | 7 | 19 | 9.6 | 0 | 1.68 | -0.3 – 3.6 | 0.393 | 19 | 9.6 | 1 | 1.3 | -0.6 – 3.3 | 0.835 |
|  | 8 | 19 | 9.6 | 0 | 0.37 | -1.6 – 2.3 | 0.883 | 17 | 19.1 | 1 | 6.3 | 4.1 – 8.4 | **0.025** |

P-values correspond to the fixed effects of the linear mixed model (LMM) and were estimated using Satterthwaite’s approximation. They test whether each term (intercept, interval, group, and their interactions) differs significantly from zero or from the reference category.

These comparisons are exploratory in nature; the absence of significant differences in some cases does not necessarily indicate a true lack of effect, as the statistical power was below 80% for some analyses.

**Supplementary Figure 2.** Comparison of PCR changes across the stages of pregnancy between normotensive women and women who developed HDP.


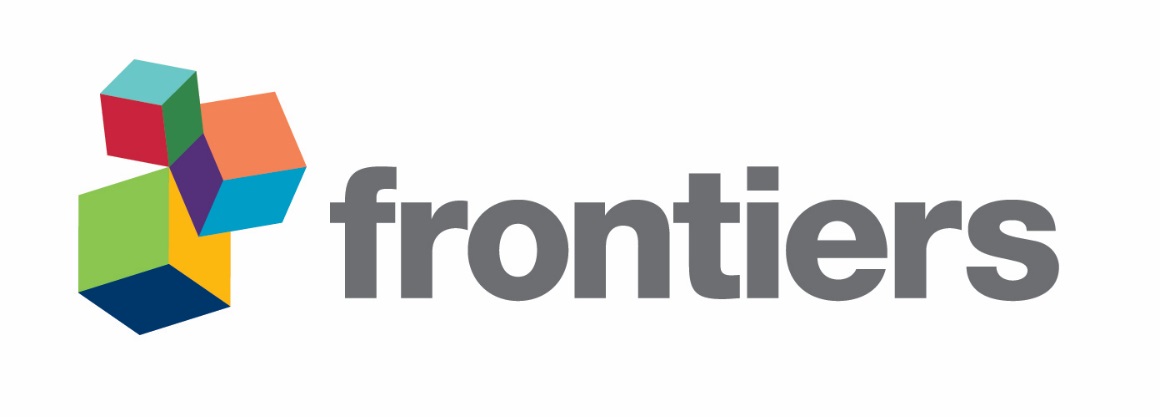

Supplement: Supplementary file 2 [file Table1.docx]
